# Translation and cross-cultural validation of a precision health tool, the Suboptimal Health Status Questionnaire-25, in Korean

**DOI:** 10.7189/jogh.12.04077

**Published:** 2022-10-01

**Authors:** Zheng Guo, Ruoyu Meng, Yulu Zheng, Xingang Li, Ziqi Zhou, Leilei Yu, Qian Tang, Ying Zhao, Monique Garcia, Yuxiang Yan, Manshu Song, Lois Balmer, Jun Wen, Haifeng Hou, Xuerui Tan, Wei Wang, Suboptimal Health Study Consortium (SHSC) and the Global Health Epidemiology Research Group (GHERG)

**Affiliations:** 1 Centre for Precision Health, Edith Cowan University, Joondalup, Western Australia, Australia; 2 School of Medical and Health Sciences, Edith Cowan University, Joondalup, Western Australia, Australia; 3 The Nathan Centre, Joondalup, Western Australia, Australia; 4 Department of Physiology, Institute of Medical Science, Jeonbuk National University Medical School, Jeonju, Korea; 5 Department of Herbology, School of Korean Medicine, Wonkwang University, Jeonbuk, Korea; 6 Department of Endocrinology, Taian City Central Hospital, Taian, China; 7 Department of Obstetrics, Tengzhou People's Central Hospital, Tengzhou, China; 8 School of Foreign Languages, Shandong First Medical University & Shandong Academy of Medical Sciences, Taian, Shandong, China; 9 Beijing Key Laboratory of Clinical Epidemiology, School of Public Health, Capital Medical University, Beijing, China; 10 School of Business and Law, Edith Cowan University, Joondalup, Western Australia, Australia; 11 School of Public Health, Shandong First Medical University & Shandong Academy of Medical Sciences, Taian, Shandong, China; 12 The First Affiliated Hospital of Shantou University Medical College, Shantou, Guangdong, China; 13 Nutrition & Health Innovation Research Institute, Edith Cowan University, Joondalup, Western Australia, Australia

## Abstract

**Background:**

Suboptimal health status (SHS) is a reversible stage between health and illness that is characterized by health complaints, low energy, general weakness, and chronic fatigue. The Suboptimal Health Status Questionnaire-25 (SHSQ-25) has been validated in three major populations (African, Asian, and Caucasian) and is internationally recognized as a reliable and robust tool for health estimation in general populations. This study focused on the development of K-SHSQ-25, a Korean version of the SHSQ-25, from its English version.

**Methods:**

The SHSQ-25 was translated from English to Korean according to international guidelines set forth by the World Health Organization (WHO) for health instrument translation between different languages. A subsequent cross-sectional survey involved 460 healthy South Korean participants (aged 18-83 years; 65.4% females) to answer the 25 questions focusing on the health perspectives of 5 domains, 1) fatigue, 2) cardiovascular health, 3) digestive tract, 4) immune system and 5) mental health. The K-SHSQ-25 was further validated using tests for reliability, internal consistency, exploratory factor analysis (EFA), and confirmatory factor analysis (CFA).

**Results:**

The version of K-SHSQ-25 achieved linguistic, cultural, and conceptual equivalence to the English version. The intraclass correlation coefficient (ICC) of test-retest reliability for individual items ranged from 0.88 to 0.99. Reliability estimates based on internal consistency reached a Cronbach’s α of 0.953; the Cronbach’s α for each domain ranged from 0.76 to 0.94. Regarding construct validity, the EFA of the K-SHSQ-25 generally replicated the multidimensional structure (fatigue, cardiovascular, digestive, immune system, and mental health) and 25 questions. The CFA revealed that the root mean square error of approximation (RMSEA), goodness-of-fit index (GFI) and adjusted goodness of fit index (AGFI) were excellent (RMSEA = 0.069<0.08, GFI = 0.929>0.90, AGFI = 0.907>0.90). The five domains of the K-SHSQ-25 showed significant correlations with each other (r = 0.59-0.81, *P*<0.001). The cut-off point of K-SHSQ-25 for SHS was determined as an SHS score of 25. The prevalence of SHS in this study was 60.0% (276/460), with 47.8% (76/159) for males and 58.5% for females (176/301).

**Conclusions:**

Our results indicate that the Korean version of SHSQ-25, K-SHSQ-25, is a transcultural equivalent, robust, valid, and reliable assessment tool for evaluating SHS in the Korean-speaking population.

The World Health Organization (WHO) defines health as “a state of complete physical, mental and social well-being and not merely the absence of disease or infirmity” [1]. With the rapidly increasing pace of life and daily stress which characterize modern society, the number of people experiencing adverse chief complaints is on the rise [2]. These conditions can be complex with a range of manifestations and are therefore generally classified as suboptimal health status (SHS) [3]. SHS has been recognized as a global public health challenge and gains increasing attention from medical professionals [2].

SHS is an intermediate and reversible stage between health and disease, indicated by health complaints, low energy, general weakness, and chronic fatigue [4]. Individuals with SHS suffer from disorders or health complaints without displaying objective signs of disease [5]. Studies have shown that modifiable behaviours such as smoking, insufficient physical activity, poor eating habits, and excessive alcohol intake influence the occurrence of SHS [6]. These behaviours are also the risk factors for non-communicable diseases (NCDs) [7]. Additionally, studies showed that SHS is associated with altered intestinal microbiota [8], telomere length [9], the mRNA expression level of glucocorticoid receptor α [10], cardiovascular health metrics [6], plasma cortisol [11], plasma catechol-amines [12], oxidative stress [13], blood transcriptome profiling [14], metabolites [15], and IgG N-glycans profiling [16-18]. Combined with the objective measures of biomarkers, SHS assessment can potentially predict early-stage NCDs like cancer [19], cardiovascular disease [20], dyslipidaemia [21], hypertension [22], metabolic syndrome [16], Parkinson’s disease [23,24], preeclampsia [25], rheumatoid arthritis [26], stroke [22], systemic lupus erythematosus [27], and type 2 diabetes mellitus [28].

To facilitate time-efficient and cost-effective SHS evaluation, we previously developed an innovative, reliable, valid, and robust assessment, the Suboptimal Health Status Questionnaire-25 (SHSQ-25). It has been validated among three major ethnic groups (African, Asian, and Caucasian) and has shown high validity and reliability for health measuring. The SHSQ-25 is a self-reported instrument, encompassing five domains with 25 items (**Figure 1**) that promises early detection and intervention of NCDs to reduce disease burden [3,29,30]. South Korea has achieved one of the world’s highest economic growth rates in recent decades and is one of the four most developed countries in Asia. The country is also heavily influenced by western cultures and lifestyles. To overcome language barriers and expand the application of the SHSQ-25 in a non-English-speaking country like Korea, it is essential to linguistically and cross-culturally translate the SHSQ-25 into a local language (e.g., Korean) and to validate the translated SHSQ-25 in the local population prior to its administration [31]. We intended to translate the SHSQ-25 in English into the Korean language and to evaluate the validity and reliability of the K-SHSQ-25 in a Korean population.

**Figure 1:**
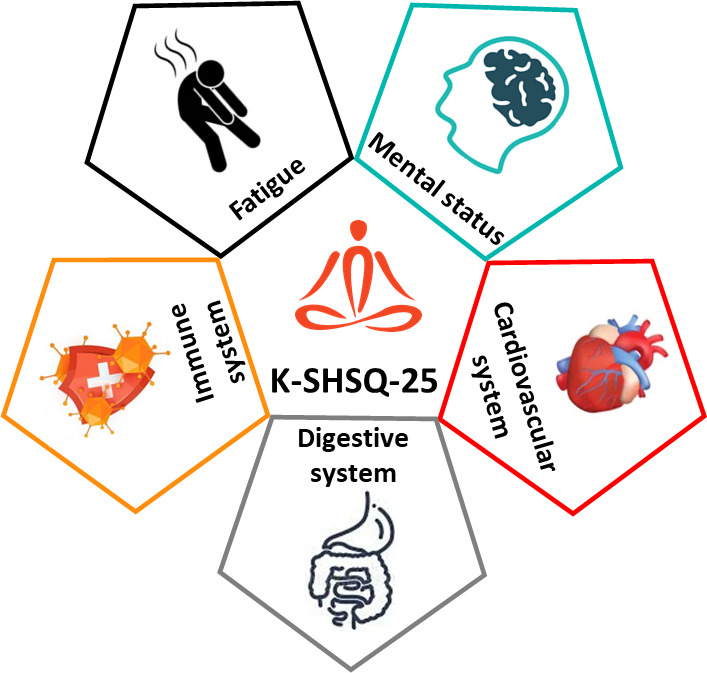
The Korean version of Suboptimal health status questionnaire-25 (K-SHSQ-25): five domains.

## METHODS

### Description of the SHSQ-25

The SHSQ-25 includes 25 items across 5 health domains: 1) fatigue, 2) cardiovascular health, 3) digestive tract, 4) immune system, and 5) mental health (Figure 1) [3]. SHSQ-25 items are scored on a 5-point Likert-type scale based on the frequency of specific complaints: 1) never or almost never, 2) occasionally, 3) often, 4) very often, and 5) always [3]. Raw SHS score of 1-5 in the questionnaire is recorded as 0-4, and the SHSQ-25 score is calculated as the sum of the scores of its 25 items [3].

### Translation

**Figure 2:**
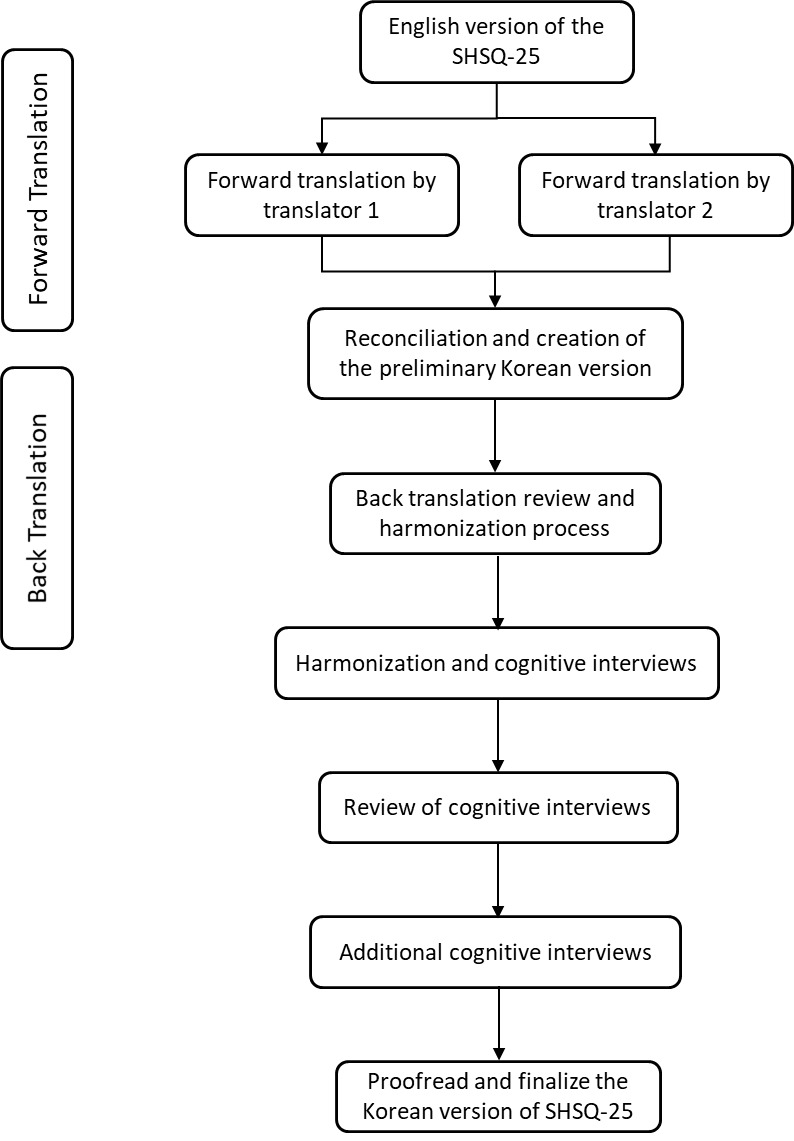
Flow chart of translation methodology.

The SHSQ-25 was translated into Korean following the WHO’s Guidelines on Translation and Adaptation of Instruments to ensure linguistic and cultural equivalence between the English version and the translated version [32]. A flowchart of the translation process is shown in **Figure 2**.

The literal forward translation of the SHSQ-25 from its source language (English) into the target language (Korean) was completed by two independent researchers familiar with the concepts covered by the SHSQ-25 and fluent in both English and Korean. The two initial translated versions were later merged into a single Korean version after achieving consensus between the two translators and further confirmation by the senior authors.

The harmonized Korean version of the SHSQ-25 was next back-translated into English by the other two bilingual Korean-English translators who were unfamiliar with the SHSQ-25. The back-translated and original versions were subsequently compared to ensure linguistic, cultural, and conceptual equivalence.

Cognitive debriefing interviews were conducted to review the clarity, cultural adequacy, acceptability, understandability, and appropriateness of the translated items in the Korean version of the SHSQ-25. Written notes were taken during the interviews to compile the feedback. Relevant modifications were then made to the translated Korean version, after which additional interviews were held to review the alterations. Items’ spelling, grammar, and formatting were proofread; K-SHSQ-25, the translated Korean version of the SHSQ-25, is presented in Appendix B in the **Online Supplementary Document**.

### Recruitment of K-SHSQ-25 survey participants

Participants were randomly recruited from a local Korean community via convenience sampling. They were required to meet the following inclusion criteria: 1) no history of somatic or psychiatric abnormalities; 2) aged over 18; and 3) no history of medication consumption in the previous 2 weeks. We excluded individuals suffering from a specific disease, e.g., diabetes, cardiovascular, respiratory, genitourinary, digestive, or circulatory disease.

### Validation

#### Reliability

Test-retest reliability between the first and second responses to the questionnaire was calculated for all items based on the intraclass correlation coefficient (ICC) [33]. An ICC below 0.50 reflected poor reliability, between 0.50 and 0.75 reflected moderate reliability, and above 0.75 reflected good reliability [33].

Internal consistency is a measurement that estimates a questionnaire’s reliability. Item internal consistency (IIC) was used to estimate the reliability of items within separate domains [34,35]. The Cronbach’s α coefficient, ranging from 0 to 1, was computed to analyse internal consistency [36]. Cronbach’s α values ranged between excellent (≥0.9), good (0.8-0.9), acceptable (0.7-0.8), questionable (0.6-0.7), poor (0.5-0.6), and unacceptable (≤0.5). A Cronbach’s α of 0.40 was suggested as the standard for supporting an IIC [37].

#### Construct validity

Exploratory factor analysis (EFA) was used to explore the underlying theoretical structure of the K-SHSQ-25. The appropriateness of collected data for EFA was measured using Bartlett’s test of sphericity and also the Kaiser-Meyer-Olkin (KMO) measure of sampling adequacy to assess factorability of the correlation matrix [38,39]. *P*<0.05 was considered statistically significant for Bartlett’s test of sphericity [38]. KMO values ranged between 0 and 1; a value of less than 0.6 was considered inadequate [39]. Maximum likelihood factors analysis with subsequent Promax rotation was used to extract the factors [40]. A factor loading of at least 0.40 was used to determine the cut-off point [41,42].

Confirmatory factor analysis (CFA) was used to assess the multidimensional structure of the K-SHSQ-25. Maximum likelihood factor analysis with subsequent Promax rotation was employed to assess goodness of fit, namely the Chi-square (χ^2^), root mean square error of approximation (RMSEA), goodness-of-fit index (GFI), and adjusted goodness-of-fit index (AGFI). RMSEA values lower than 0.08, and GFI and AGFI values greater than 0.90, denoted a reasonable fit of the K-SHSQ-25 [43,44]. Pearson’s correlation analysis was performed to measure the correlations among individual questionnaire domains (fatigue, cardiovascular health, digestive tract, immune system, and mental health).

### Statistical analyses

The distribution of the scores of K-SHSQ-25 was tested for normality using the Kolmogorov–Smirnov test. Statistical analyses were conducted in SPSS version 27.0 for Windows (IBM Corp., Armonk, NY); CFAs were performed in AMOS version 28.0 for Windows (IBM Corp., Armonk, NY). The cut-off point of SHS in the Korean population was determined as one-tailed 90% upper limit of K-SHSQ-25 scores [45]. All reported *P*-values were two-tailed, and *P*<0.05 was considered statistically significant.

## RESULTS

### Characteristics of participants

**Table 1** lists participants’ demographics. A total of 500 questionnaires were distributed during the 10-month study period; 460 complete responses were received (92% response rate). Most participants (n = 301; 65.4%) were women and 159 (34.6%) were men, with a mean age of 36.0 (SD = 11.9 years; range = 18-83). Most participants held a university degree (72.6%).

**Table 1:** Characteristics of Questionnaire Respondents (n = 460)

Variable	N	%	Median SHS scores	Mean SHS scores ± SD	*P*
**Sex**					
Female	301	65.4	27	29 ± 16	0.025*
Male	159	34.6	25	26 ± 11	
**Age**					
18-30	161	35.0	26	27 ± 16	0.263†
31-40	170	37.0	27	30 ± 15	
41-50	66	14.3	25	27 ± 13	
51-60	51	11.1	26	25 ± 8	
>60	12	2.6	25	24 ± 5	
**Highest level of education**					
Illiteracy/primary	41	8.9	25	26 ± 13	0.108†
Middle school	85	18.5	25	25 ± 15	
College/university	334	72.6	26	29 ± 14	

SHS – suboptimal health status, SD – standard deviation, N – number

*Mann-Whitney U test.

†Kruskal-Wallis test.

### Test-retest reliability

Of the 50 respondents included in the test-retest study, 44 completed the second questionnaire (88%), which was administered seven days after the first test. The participants were 23 (52.3%) men and 21 (47.7%) women with a mean age of 35.8 (SD=11.3 years). The median ICC between the test and retest ratings for each individual item was 0.95 (95% confidence interval (CI) = 0.88-0.99). ICCs for the overall SHS and the individual domains of SHS are presented in **Table 2**.

**Table 2:** Results for 5 Subscales of the K-SHSQ-25

Domain	N of items	Mean ± SD	Cronbach’s α	IIC	ICC* (95% CI)
Fatigue	9	11.97 ± 5.64	0.88	0.86-0.88	0.94 (0.89-0.97)
Mental status	7	7.74 ± 5.20	0.94	0.92-0.94	0.95 (0.91-0.97)
Cardiovascular system	3	2.39 ± 1.91	0.76	0.62-0.75	0.91 (0.88-0.93)
Digestive system	3	2.77 ± 1.97	0.79	0.65-0.74	0.86 (0.75- 0.93)
Immune system	3	2.86 ± 1.74	0.77	0.41-0.58	0.99 (0.98-0.99)
Total	25	27.72 ± 14.45	0.95	–	0.98 (0.97-0.99)

N – number, SD – standard deviation, CI – confidence interval, IIC – item internal consistency, ICC – intraclass correlation coefficient

*Results from the 2-way mixed model.

### Internal consistency

The internal consistency of individual domains and composite scores are summarized in **Table 2**. Internal consistency for the overall SHS was excellent (Cronbach’s α = 0.953). The IIC for each SHS domain was 0.76 or higher, indicating that all items met the standard of 0.40 for sound internal consistency.

### Exploratory factor analysis

The KMO measure was 0.954 and Bartlett’s test of sphericity was significant (χ^2^(300) = 7502.658; *P*<0.001), showing that the study sampling was adequate to perform EFA. The EFA of the K-SHSQ-25 generally replicated the multidimensional structure (fatigue, cardiovascular, digestive, immune system, and mental health; Table S1 in the **Online Supplementary Document**) and 25 questions from the English version of SHSQ-25 (Appendix A in the **Online Supplementary Document**) with factors interpreted when loadings were greater than 0.40; there was no cross-loading.

### Confirmatory factor analysis

**Figure 3:**
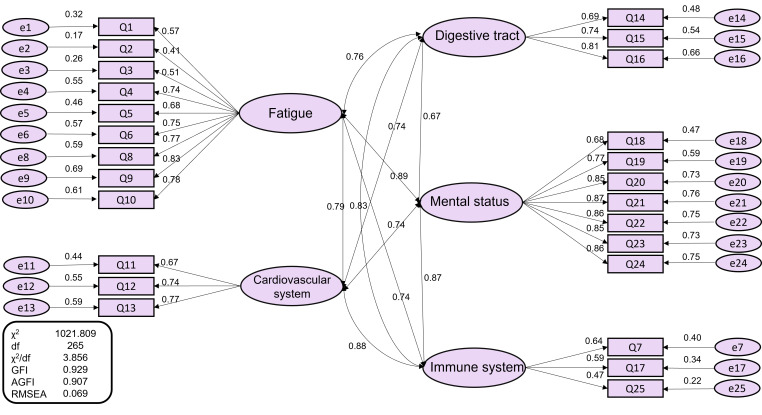
Confirmatory analysis of the 5 domains and 25 elements of the SHSQ-25. χ^2^ – chi-square, df – degree of freedom, RMSEA – root mean square error of approximation, GFI – goodness-of-fit index, AGFI – adjusted goodness-of-fit index.

CFA was performed to confirm the K-SHSQ-25’s factor structure. The results of CFA showed no modification from EFA. Goodness of fit, assessed using appropriate indices (i.e., RMSEA, GFI, and AGFI), and the path diagram were presented in **Figure 3**. The CFA based on five intercorrelated factors showed a reasonable fit of the data to the factor structure: RMSEA = 0.069 (95% CI = 0.065-0.073); GFI = 0.929; AGFI = 0.907.

**Figure 4:**
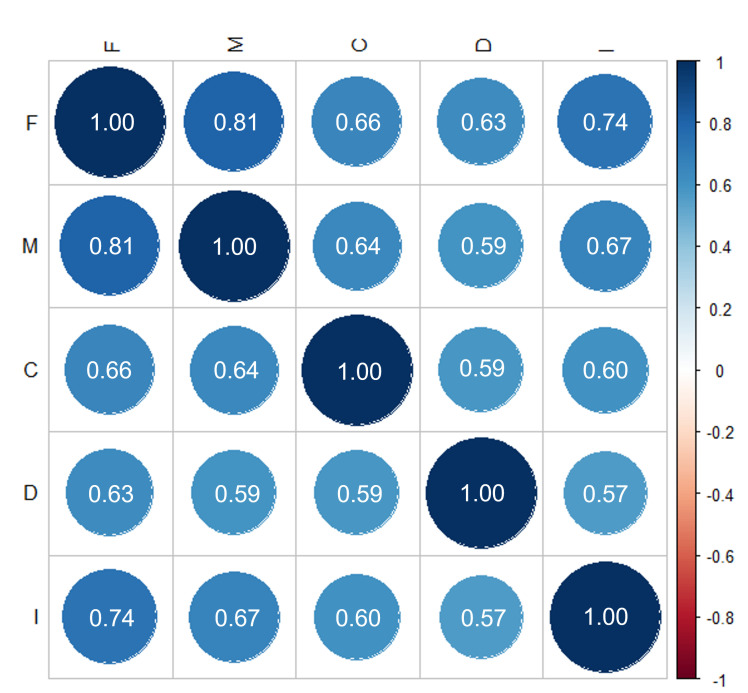
The correlation coefficients in independent domains. F – fatigue, M – mental status, C – cardiovascular system, D – digestive system, I -immune system.

Pearson’s correlation analysis was used to evaluate the correlations among individual domains. The five domains of the K-SHSQ-25 (i.e., fatigue, cardiovascular system, digestive tract, immune system, and mental health) were all found significantly correlated with each other (*P*<0.001) with the Pearson’s correlation coefficient (r) ranging from 0.57-0.81 (**Figure 4**).

### Determination of cut-off point for K-SHSQ-25

**Figure 5:**
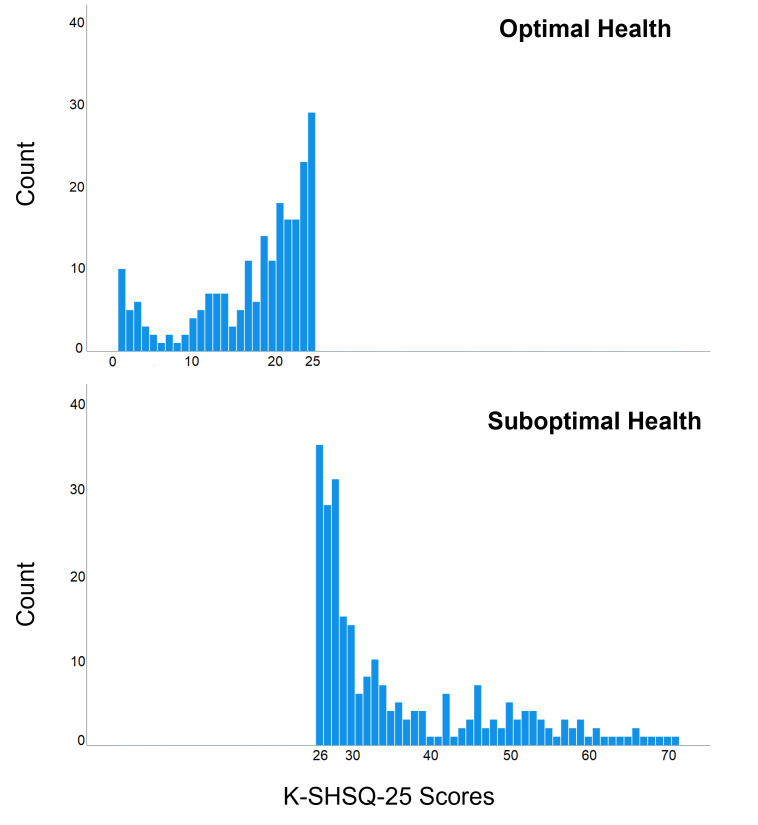
Frequency distribution of K-SHSQ-25 scores for the optimal and suboptimal health groups.

The result of Kolmogorov–Smirnov test showed that the K-SHSQ-25 scores were not normally distributed. Thus, the cut-off point should be determined as the one-tailed 90% upper limit of K-SHSQ-25 scores, i.e., a SHS score of 25. A K-SHSQ-25 SHS score >25 represented “suboptimal health”, while a score of ≤25 indicated “optimal health” (**Figure 5**). In this study, the prevalence of SHS was 60.0% (276/460), and it is higher among females (58.5%; 176/301) than that in males (47.8%; 76/159).

## DISCUSSION

SHS is “an overall physical status between health and illness characterized by the perception of health complaints, chronic fatigue, and a constellation of physical symptoms such as the cardiovascular system, the digestive system, the immune system, and mental status” [4,46]. Although SHS is not a disease state, it commonly represents the period preceding the occurrence of clinical manifestations of diseases.

The SHSQ-25 is a reliable and valid 5-point Likert-type scale and validated in three major ethnic groups: African [29], Asian [3], and Caucasian [20]. As an established tool from the perspective of precision healthcare, the SHSQ-25 can be applied in both health care and community settings to identify individuals experiencing poor health condition without a diagnosable disease. This study aimed to produce a Korean version of the questionnaire suitable for research and clinical settings with a Korean-speaking population.

Following a rigorous and cross-cultural translation process including forward and back-translation, K-SHSQ-25, the Korean version of the SHSQ-25, was found to be linguistically, culturally, and conceptually equivalent to the English version of SHSQ-25. No major difficulties were encountered during the translation process. The structure and all questions from the English version of SHSQ-25 were maintained in the K-SHSQ-25.

The pilot survey results suggested that the K-SHSQ-25 is a reliable, robust, and valid instrument to identify SHS among a Korean-speaking population. The K-SHSQ-25 demonstrated adequate test-retest reliability (ICC = 0.95; 95% CI = 0.88-0.99) and internal reliability (Cronbach’s α = 0.95) that corresponded to values values observed with the English version of SHSQ-25 (ICC = 0.93; 95% CI = 0.91-0.95; Cronbach’s α = 0.93) [3]. Additionally, the 92% response rate indicated that the K-SHSQ-25 is readily implementable among South Korean and that participants were able to accurately respond to the questions in the K-SHSQ-25.

To assess construct validity, we conducted EFA and CFA on the five domains of the SHSQ-25. Results of KMO and Bartlett’s test, which are pre-requisites for performing EFA, confirmed the sampling adequacy in this study. In the K-SHSQ-25, EFA extracted five domains (i.e., fatigue, cardiovascular system, digestive tract, immune system, and mental health) that are consistent with the English version of SHSQ-25. Results of CFA revealed a good fit of the data based on the RMSEA (0.069; 95% CI = 0.065-0.073), GFI (0.929), and AGFI (0.907), all of which reached the respective standards. Our findings further demonstrated that the five domains and the items in each domain are consistent with the English version of the SHSQ-25 [3]. Pearson’s correlation analysis revealed strong correlations among each domain of the K-SHSQ-25: all correlation coefficients exceeded 0.57 and were statistically significant.

Although the concept of SHS, which was derived from traditional Chinese medicine, is applicable to South Korea, an eastern Asian country. Adding to the English [3], Chinese [45,47] and Russian [20] versions of SHSQ-25, this present study generated the K-SHSQ-25, a Korean version of the SHSQ-25. The results showed that the K-SHSQ-25 has been successfully cross-culturally translated and can be applied as a valid, reliable instrument to assess SHS among Korean populations.

This study has limitations needed to be addressed; it was conducted in a single centre. Future studies should involve multicentre replication with a larger sample.

## CONCLUSIONS

The SHSQ-25 was successfully translated into Korean without losing any properties of the English version. The K-SHSQ-25 was further validated as a reliable health measure to evaluate SHS in a Korean-speaking population. Development of the K-SHSQ-25 represents an example of overcoming language barriers and enables comparisons when using the same instrument in multiple languages. The K-SHSQ-25 is a useful tool to evaluate and monitor individuals’ health status. We thus recommend the K-SHSQ-25 as a cost-effective and time efficient tool in clinical settings and community-based health surveys in the Korean population.

## Additional material


Online Supplementary Document
