# Supplementary material for: Translation and cross-cultural validation of a precision health tool, the Suboptimal Health Status Questionnaire-25, in Korean
Source: J Glob Health. 2022 Oct 1;12:04077. doi: 10.7189/jogh.12.04077 (PMC9526479; doi:10.7189/jogh.12.04077)
Supplement: Online Supplementary Document [file jogh-12-04077-s001.pdf]

ONLINE SUPPLEMENTARY DOCUMENT

Title: Translation and Cross-Cultural Validation of a Precision Health Tool, the Suboptimal Health Status Questionnaire-25 (SHSQ-25), in Korean

Authors: Zheng Guo, Ruoyu Meng, Yulu Zheng, Xingang Li, Ziqi Zhou, Leilei Yu, Qian Tang, Ying Zhao, Monique Garcia, Yuxiang Yan, Manshu Song, Lois Balmer, Jun Wen, Haifeng Hou, Xuerui Tan, Wei Wang, on behalf of the Suboptimal Health Study Consortium (SHSC) and the Global Health Epidemiology Research Group (GHERG)

Table S1. Loadings of Variables on Factors Emerging from Factor Analysis

Item		Factors				
	**Abbreviated item-label**	**Fatigue**	**Immune System**	**Cardiovascular System**	**Digestive Tract**	**Mental Status**
**Q1**	Exhaustion	0.683				
**Q2**	Chronic fatigue	0.777				
**Q3**	Lethargy when working	0.717				
**Q4**	Headache	0.633				
**Q5**	Dizziness	0.685				
**Q6**	Aching or tired eyes	0.614				
**Q7**	Sore throat		0.766			
**Q8**	Muscle or joint stiffness	0.522				
**Q9**	Ache in shoulder/neck/waist	0.598				
**Q10**	Heavy feeling in legs	0.546				
**Q11**	Breathlessness			0.623		
**Q12**	Chest congestion			0.638		
**Q13**	Heart palpitations			0.605		
**Q14**	Poor appetite				0.753	
**Q15**	Upset stomach				0.642	
**Q16**	Indigestion				0.683	
**Q17**	Cold intolerance		0.605			
**Q18**	Difficulty failing asleep					0.531
**Q19**	Waking up during the night					0.640
**Q20**	Impaired short-term memory					0.810
**Q21**	Inability to respond quickly					0.765
**Q22**	Difficulty concentrating					0.754
**Q23**	Distracted for no reason					0.766
**Q24**	Nervous or jittery					0.754
**Q25**	Frequently catch colds		0.754			

Appendix

The English version of Suboptimal health status questionnaire-25 (SHSQ-25) (A), and the Korean version of Suboptimal health status questionnaire-25 (KSHSQ-25) (B).

A

How often is it, that you (your)	Never or almost 1	Occasionally 2	Often 3	Very often 4	Always 5
1. were exhausted without greatly increasing your physical activity	◯	◯	◯	◯	◯
2. fatigue could not be substantially alleviated by rest	◯	◯	◯	◯	◯
3. were lethargic when working	◯	◯	◯	◯	◯
4. suffered from headaches	◯	◯	◯	◯	◯
5. suffered from dizziness	◯	◯	◯	◯	◯
6. eyes ached or were tired	◯	◯	◯	◯	◯
7. suffered from a sore throat.	◯	◯	◯	◯	◯
8. muscles or joints felt stiff	◯	◯	◯	◯	◯
9. have pain in your shoulder/neck/waist	◯	◯	◯	◯	◯
10. have a heavy feeling in your legs when walking	◯	◯	◯	◯	◯
11. feel out of breath while sitting still	◯	◯	◯	◯	◯
12. suffered from chest congestion	◯	◯	◯	◯	◯
13. were bothered by heart palpitations	◯	◯	◯	◯	◯
14. appetite is poor	◯	◯	◯	◯	◯
15. suffered from heartburn	◯	◯	◯	◯	◯
16. suffered from nausea	◯	◯	◯	◯	◯
17. could not tolerate the cold	◯	◯	◯	◯	◯
18. had difficulty falling asleep	◯	◯	◯	◯	◯
19. had trouble with waking up during the night	◯	◯	◯	◯	◯
20. had trouble with your short-term memory	◯	◯	◯	◯	◯
21. could not respond quickly	◯	◯	◯	◯	◯
22. had difficulty concentrating	◯	◯	◯	◯	◯
23. were distracted for no reason	◯	◯	◯	◯	◯
24. felt nervous or jittery	◯	◯	◯	◯	◯
25. caught a cold in the past 3 months	◯	◯	◯	◯	◯

B

얼마나 자주, 너 (너의)	전혀 없었다 1	거의없었다 2	그렇다 3	자주 그렇다 4	거의 항상 그렇다 5
1. 귀하는 피곤하십니까 운동량이 현저히 증가되지 않는 경우	◯	◯	◯	◯	◯
2. 귀하는 휴식 후 피로가 풀릴 수 있습니까	◯	◯	◯	◯	◯
3. 귀하는 일할 때 졸리거나 권태로운 현상이 있습니까	◯	◯	◯	◯	◯
4. 두통	◯	◯	◯	◯	◯
5. 머리가 어지럽다	◯	◯	◯	◯	◯
6. 눈이 쓰리고 탱탱하다	◯	◯	◯	◯	◯
7. 인후통	◯	◯	◯	◯	◯
8. 근육과 관절은 항상 경직되거나 뻣뻣함을 느낀다	◯	◯	◯	◯	◯
9. 어깨 목 또는 허리가 시큰거리다	◯	◯	◯	◯	◯
10. 걸을 때 다리가 무겁다	◯	◯	◯	◯	◯
11. 숨을 죽일 때 숨이 가쁘다	◯	◯	◯	◯	◯
12. 가슴이 답답하다	◯	◯	◯	◯	◯
13. 속이 떨리다	◯	◯	◯	◯	◯
14. 식욕 감퇴	◯	◯	◯	◯	◯
15. 속이 안 좋다	◯	◯	◯	◯	◯
16. 소화 불량	◯	◯	◯	◯	◯
17. 저열 혹은 추위를 탄다	◯	◯	◯	◯	◯
18. 잠들기 힘들다	◯	◯	◯	◯	◯
19. 꿈이 많거나 놀라 깨기 쉽다	◯	◯	◯	◯	◯
20. 기억력이 감퇴하다	◯	◯	◯	◯	◯
21. 순발력이 떨어지다	◯	◯	◯	◯	◯
22. 집중력이 떨어지다	◯	◯	◯	◯	◯
23. 이유 없이 정신이 사납다	◯	◯	◯	◯	◯
24. 긴장, 불안	◯	◯	◯	◯	◯
25. 귀하는 최근 3 개월 동안 감기에 걸린 적이 있습니까	◯	◯	◯	◯	◯
